# Anode Modification with Fe_2_O_3_ Affects the Anode Microbiome and Improves Energy Generation in Microbial Fuel Cells Powered by Wastewater

**DOI:** 10.3390/ijerph20032580

**Published:** 2023-01-31

**Authors:** Dawid Nosek, Tomasz Mikołajczyk, Agnieszka Cydzik-Kwiatkowska

**Affiliations:** 1 Department of Environmental Biotechnology, University of Warmia and Mazury in Olsztyn, Słoneczna 45 G, 10-709 Olsztyn, Poland; 2 Department of Chemistry, University of Warmia and Mazury in Olsztyn, plac Łódzki 4, 10-721 Olsztyn, Poland

**Keywords:** MFC, iron, anode modification, internal resistance, microbiome

## Abstract

This study investigated how anode electrode modification with iron affects the microbiome and electricity generation of microbial fuel cells (MFCs) fed with municipal wastewater. Doses of 0.0 (control), 0.05, 0.1, 0.2, and 0.4 g Fe_2_O_3_ per the total anode electrode area were tested. Fe_2_O_3_ doses from 0.05 to 0.2 g improved electricity generation; with a dose of 0.10 g Fe_2_O_3_, the cell power was highest (1.39 mW/m^2^), and the internal resistance was lowest (184.9 Ω). Although acetate was the main source of organics in the municipal wastewater, propionic and valeric acids predominated in the outflows from all MFCs. In addition, Fe-modification stimulated the growth of the extracellular polymer producers *Zoogloea* sp. and *Acidovorax* sp., which favored biofilm formation. Electrogenic *Geobacter* sp. had the highest percent abundance in the anode of the control MFC, which generated the least electricity. However, with 0.05 and 0.10 g Fe_2_O_3_ doses, *Pseudomonas* sp., *Oscillochloris* sp., and *Rhizobium* sp. predominated in the anode microbiomes, and with 0.2 and 0.4 g doses, the electrogens *Dechloromonas* sp. and *Desulfobacter* sp. predominated. This is the first study to holistically examine how different amounts of Fe on the anode affect electricity generation, the microbiome, and metabolic products in the outflow of MFCs fed with synthetic municipal wastewater.

## 1. Introduction

Microbial fuel cells (MFCs) convert energy from biomass into electricity using electroactive bacteria (electrogens, EEs). To date, most research has been conducted at the laboratory scale because of the cells’ low power output, making it challenging to transfer MFC technology to commercial-scale systems [1]. As far as the authors know, the largest MFC tested to date had a volume of 1200 L [2].

The most important factor for the efficiency of MFCs is the electrode material. Carbon materials that possess good electrical conductivity, such as carbon felt (CF), carbon fabric, or carbon paper, are most commonly used [3]. In recent years, the focus has been on improving these materials by coating their surfaces with metal oxides, which increases the surface roughness, thereby improving the adhesion of bacteria and increasing electron transfer at the anode [4,5]. Iron oxide, for example, improves the power output of MFCs by stimulating the activity of microorganisms and by improving electrical conductivity. Iron oxide (III) is an attractive material for anode electrode modification because of its properties to improve the surface structure of the anode and generate electrical energy in the MFC. Studies report that modifying the anode electrode with Fe_2_O_3_ changes the surface from smooth to rough, improving the microorganisms’ attachment [6]. Additionally, the hydrophobic carbon surface can be hydrophilized by coating with Fe_2_O_3_ due to the hydrogen bond formed between the Fe_2_O_3_ oxygen and the water molecule [7]. The advantages of choosing this material are its easy availability, chemical stability, and low cost. Furthermore, insoluble Fe_2_O_3_ has a high affinity for type C cytochromes (OmcA and MtrC) [8], which influences the increased electron transfer; thus, it is often used as a modifier in MFC layouts. Studies show that the presence of Fe(III) oxide increases the proportion of electroactive bacteria such as Pseudomonas sp. and *Geobacter* sp. on the anode [9]. Yamashita et al. [10] tested a flame-oxidized (FO) stainless steel (SS) anode in a single-chamber MFC compared to SS and CC anodes without treatment. The tests showed that the flame oxidation produced spots on the anode consisting mainly of Fe_2_O_3_, which increased the power density by 184 and 24% over untreated SS and CC. *Geobacter* sp. bacteria were more numerous on the FO–SS anode (8.8–9.2%) than on SS and CC (0.7–1.4%). Another study tested the addition of Fe_2_O_3_ to sediment MFC (SMFC). The TOC and DOC removal rates were 2.6 and 1.82 times higher in SMFC with iron oxide (III), respectively than in open-cycle reactors without the addition of Fe(III). The presence of Fe(III) increased the proportion of *Pseudomonas* sp. and *Desulfobacterium* sp. in biofilm about two-fold [11]. In another study, a composite anode SS with ultracapacitor powdered activated carbon (UAC) was tested with Fe_2_O_3_. The MFC with Fe_2_O_3_ showed a faster start-up time than the anode without the addition of Fe_2_O_3_. It was found that the Fe_2_O_3_-modified anode showed the highest repeatable voltage of 550 mV. Tafel’s electrochemical extrapolation technique showed that MFC anode oxidation activation was stronger when Fe_2_O_3_ was added. The authors attribute the increase in kinetic activity to the facilitated extracellular electron transfer from the cell to the bacteria, showing that the rate of anode electron transfer can be increased by the addition of Fe_2_O_3_ [12]. Sayed et al. [13] found that a carbon cloth (CC) anode covered with Fe nanostructures had a lower potential than an unmodified anode (−0.01 and 0.16 V vs. Ag/AgCl, respectively). The high roughness and the nano-layer structure of the Fe-CC anode provided a greater surface area for growth and electron transfer between the microorganisms and the anode surface, increasing the MFC’s maximum power and current density.

Similarly, Sekar et al. [14] synthesized copper-doped iron oxide nanoparticles with the use of *Amaranthus blitum* phytochemicals and found that they showed good hydrophilic properties on the anode surface. Modification of the anode with copper-doped iron oxide increased peak power density to 161.5 mW/m^2^, decreased ohmic resistance by 98%, decreased charge transfer resistance by 95%, and increased the power density of the cell 1.3 times. Wang et al. [15] used anodes modified with α-Fe_2_O_3_ in conjunction with polyelectrolytes for energy generation in MFCs. The highest power density was observed in an MFC with a CC anode covered with four double layers of polydimethyl diallyl ammonium chloride, poly sodium-p-styrene sulfonate, and one layer of α-Fe_2_O_3_. The use of this anode ensured the lowest internal resistance of the MFC, and the high roughness and large surface area of the anode were beneficial for microbial growth.

In MFCs, iron-reducing EEs, such as *Geobacter* sp. and *Shewanella* sp., play significant roles. As iron compounds are insoluble at pH 7–8, these bacteria reduce Fe either via direct contact with their outer membranes’ cytochromes or with conductive pili [16]. For example, in an MFC with a CF anode modified with graphene oxide/Fe_2_O_3_ that was supplied with pure acetate, the maximum stable voltage was 590 ± 5 mV, and the presence of iron increased the abundance of EEs belonging to *Desulfovibrio* sp. [17].

Fe addition can not only increase the output of MFCs, but it can also improve wastewater treatment. In a bioelectrochemical system, the use of Fe-carbon electrodes increased the activities of denitrifying enzymes such as nitrite reductase, nitric oxide reductase, and nitrous oxide reductase [18]. Dosing Fe(III) during wastewater treatment favors the removal of micropollutants via the adsorption of compounds on the surface of iron sulfide (FeS), followed by their biodegradation [19]. Moreover, Fe addition also prevents methane production under redox conditions [20], a common problem in MFCs.

The effect of anode modification with Fe depends on the anode material. Mohamed et al. [6] modified CF, CC, and graphite (G) anodes with Fe and found that the presence of Fe improved the wettability of the electrode surface, the rate of degradation of organic compounds and the adhesion of microorganisms to the electrode surface, and it decreased the electron transfer resistance. After modification, the power generated in the MFC increased by 385%, 170%, and 130% for the CF-, CC-, and G-based electrodes, respectively. In addition, in the MFCs with modified electrodes, over 80% of organic compounds were removed from the effluent.

Although adding Fe promotes the formation of the anode’s biofilm and accelerates MFC start-up, high doses of Fe may reduce cell efficiency. Zheng et al. [21] investigated how different doses of Fe_3_O_4_ in a medium affect energy generation and the anodic microbiome in an MFC. A dose of 4.5 g/L provided the highest power (391.11 ± 9.4 mW/m^2^) and the highest share of electroactive bacteria of the genus *Geobacter* sp. in the microbiome. In a different study, 100 µM FeSO_4_ improved the performance of an MFC so that it produced a maximum voltage of 0.55 V, but higher doses reduced the voltage to 0.47 V. Iron salts at a dose of 200 µM decreased *Geobacter* sp. abundance from 49.3% to 24.4% [22]. Similarly, another study found that 200 µM Fe(III) increased an MFC’s electrochemical activity and current efficiency and decreased the anode overpotential. However, excess amounts of Fe(III) (1000 and 2000 µM) competed with the anode for electrons and inhibited the electrochemical activity of biofilms, thus lowering the power density [23]. Mechanistic investigations showed that Fe_3_O_4_ increased the conductivity of the fermented sludge, providing a better conductive environment for the anaerobic microbes. A Fe(II)/Fe(III) redox cycle was present in the fermentation system with Fe_3_O_4_, which likely increased electron transfer (ET) efficiency [24].

An important aspect of the large-scale use of MFC is the durability of the anode electrodes. Since they come into contact with the water environment and microorganisms, which may cause them to swell, materials with hydrophobic properties should be used. The surface of the anode electrodes should be rough enough to increase the adhesion of organisms, but not too rough so as not to cause the growth of pollutants resulting from the prolonged use of the electrodes [25]. Yaquoob et al. [26] studied the durability of composite graphene oxide (GO) electrodes made from biomass and the same GO with TiO_2_. The longevity of the fabricated electrodes was 85 days. The increasing voltage on both electrodes indicated stable colonization of bacterial species on the surface of the anodes. Later, the voltage suddenly dropped due to cell death. The second cycle with fresh inoculum also showed an increasing voltage trend to 170 mV, which was due to the fresh inoculum source and organic substrate enhancing the bacterial respiration process to actively oxidize the substrate. The introduction of TiO_2_ nanoparticles increased the lifetime of the composite anode, resulting in better interaction between bacterial cells and anode, smooth anode surface, and higher anode conductivity. In addition, a visualization test was performed after the completion of the reaction (85 days), and the electrodes were found to be mechanically stable and in excellent condition. Gnana Kumar et al. [27] tested a CC composite anode with reduced graphene oxide (rGO), poly(3,4-ethylenedioxythiophene) (PEDOT), and iron oxide (Fe_3_O_4_) nanorods. The authors analyzed electrode lifetime in an open circuit voltage (OCV) as a function of time under a constant load mode with an external resistance of 510 Ω. Bare CC had a lower lifetime due to the reduction of the exposed surface after repeated cycles, due to the large number of water particles on the surface as well as the bare mass CC. The composite of rGO and Fe_3_O_4_ prevented shrinkage of the active sites during repeated cycles and provided rapid stability of rGO/Fe_3_O_4_/CC parameters. Among the tested catalysts, the rGO/PEDOT/Fe_3_O_4_ composite was characterized by high physical and electrochemical strength and high electrical conductivity, which effectively prevented the destruction of the electrocatalytic activities due to strong π-π interactions and Fe-S coordination bonds between the active carbon support and the conductive polymer and the conductive polymer and Fe_3_O_4_ nanorods, respectively. The maximum OCV of 0.45 V was maintained for 200 h, and the concrete OCV was maintained for 600 h in three cycles.

Introducing Fe into an MFC system, as in the studies cited above, requires regular monitoring of Fe concentration in the effluent and carries the risk of elevated Fe concentrations in the MFC effluents. In contrast, covering the anode with Fe not only ensures direct contact between the microorganisms and Fe, but the fact that the biofilm covers the anode minimizes the risk of Fe contamination of the wastewater.

Therefore, the objective of this study was to holistically determine the influence of an MFC’s anode modification with different doses of Fe_2_O_3_ on the power generation, the microbiome, and the conversion of organic matter. The use of a low-cost method for anode modification and the use of wastewater make this proposed solution economical, thus increasing its potential for implementation. Furthermore, the molecular results obtained greatly expand the knowledge of which microorganisms play a key role in biofilm formation and electricity generation when Fe is present on the anode.

## 2. Materials and Methods

### 2.1. Experimental Set-Up

The dual-chamber MFCs were made of plexiglass. The anode and cathode chambers had active volumes of 2 L. An 8 × 8 cm Nafion 117 proton-exchange membrane (PEM) (Dupont) was used as a separator between the chambers. Before the membranes were placed in the MFCs, they were soaked in acetone for 15 min, then rinsed in distilled water, soaked in 1M HCl for 30 min, and rinsed again with distilled water. Because of membrane clogging, the membranes were cleaned in 1M HCl and rinsed with distilled water once per week (for details, see Nosek and Cydzik-Kwiatkowska [28]).

The anodes were made of carbon felt (10 × 20 × 0.3 cm) connected to a stainless-steel wire. Before use, the carbon felt was sonicated in an ultrasonic bath (InterSonic, 15 min) to remove impurities. An MFC with an unmodified anode was used as a control (MFC_control_). In the remaining MFCs, Fe_2_O_3_ was deposited on the anode in doses of 0.05 g (MFC_0.05Fe_, 1.25 g Fe_2_O_3_/m^2^), 0.1 g (MFC_0.1Fe_, 2.5 g Fe_2_O_3_/m^2^), 0.2 g (MFC_0.2Fe_, 5 g Fe_2_O_3_/m^2^), and 0.4 g (MFC_0.4Fe_, 10 g Fe_2_O_3_/m^2^). For deposition, Fe_2_O_3_ (Chempur) was suspended in 100 mL of distilled water in an ultrasonic bath for 15 min. Next, the anode was placed in a crystallizer, quenched with a Fe_2_O_3_ slurry, autoclaved (121 °C, 1.1 Bar, Classic Prestige Medical 210001), and dried at 80 °C. 

The anode chambers were inoculated with 100 mL of a 1:1 (v/v) mixture of fermentation sludge from the municipal wastewater treatment plant in Olsztyn (Poland) and a laboratory methane fermentation reactor. The MFCs were supplied with synthetic municipal wastewater [29], and sodium acetate was used as a source of organics in the amount of 400 mg COD/L. The anode chamber was sealed to prevent air access, and the chamber contents were mixed at a speed of 100 rpm/min. The cathode chamber was aerated by an air diffuser (20 mL/min). The composition of the catholyte was 75 mL of phosphate buffer and 3 g of NaCl in 2 L of distilled water, and it was replaced once per week. 

Initially, the MFCs were left for 3 days in open circuit mode to adapt the biomass to the environmental conditions in the MFCs and to support microbial colonization of the electrodes [30]. After 3 days, the contents of the anode chambers were replaced with fresh portions of wastewater. After this time, the MFCs were operated with an external resistance of 1200 Ω. This value was chosen based on preliminary experiments [31]. The operational cycle of the MFCs was 48 h; after this time, half of the anode chamber volume was replaced. The experiment was conducted for 18 cycles (36 days), and stable MFC operation was observed after 5 cycles. In the effluent, the concentration of COD, NH_4_-N, volatile fatty acids (VFAs) [32], pH, and alkalinity (TitroLine, Donserv) were determined. The VFA composition was determined using a Varian CP-3800 chromatograph [33]. For spectroscopic characterization of the surfaces of pure carbon felt after sonication in the ultrasound batch and carbon felts modified with different doses of Fe_2_O_3_, a Quanta FEG 250 Scanning Electron Microscope (SEM) equipped with Bruker XFlash 6010 Energy-dispersive X-ray spectrometer (EDX) was used.

### 2.2. Electrochemical Analyses

The polarization and power curves were determined according to Watson and Logan [34] using a True-RMS multimeter, varying the external resistance of the cell in the range of 75–7200 Ω. Voltage changes were recorded every minute using a 6600 Counts PC-LINK data acquisition unit. The current was calculated from the external resistance using Ohm’s law. Cyclic voltammetry (CV) was performed with a three-electrode system: an anode as a working electrode, a platinum countercurrent electrode, and an Ag/AgCl reference electrode with a constant potential of 0.197 mV (Gammry Instrument Interface 1010E). CV measurements were conducted at a scan rate of 20 mV/s. The electrochemical behavior of the biofilm–anode system was tested in the 14th cycle of MFC operation, 25 h after reactor feeding (stable current generation) with the catholyte present in the cathode chamber. The MFCs were operated, and the measurements were performed at room temperature.

### 2.3. Molecular Analyses

Genomic DNA was extracted from 100 µg of inoculum and biomass from the anode surface using a FastDNA SPIN Kit for Soil (MP Biomedicals). Samples from the anode surface were collected in the experiment’s 3rd, 10th, and 18th cycles. The purity and concentration of the DNA were measured using a NanoDrop spectrometer (Thermo Scientific). The DNA was amplified using a 515F/806R primer set (5′-GTGCCAGCMGCCGCGGTAA-3′/5′-GGACTACHVGGGTWTCTAAT-3’) targeting the hypervariable V4 region of bacterial and archaeal 16S rDNA genes [35]. The amplicons were sequenced using the MiSeq platform (Illumina) at Research and Testing Laboratory (USA). The reads were analyzed bioinformatically [36] and deposited in the NCBI Sequence Read Archive (BioProject PRJNA822890).

### 2.4. Statistical Analyses

The results from each MFC’s last five cycles of operation were statistically analyzed (*p* < 0.05 considered significant, Statistica 13.3, StatSoft). One-way analysis of variance (ANOVA) was used, followed by Tukey’s test (HSD). For statistical and metagenomic analysis of microbiome data, MicrobiomeAnalyst [37,38] was used (*p* < 0.05). Due to the fact that, in complex microbial communities, bacteria with a low abundance may be of great importance, the number of reads was not normalized before the calculation of diversity indices [39].

## 3. Results and Discussion

### 3.1. Analysis of the Electrode Surface

**Figure 1:**
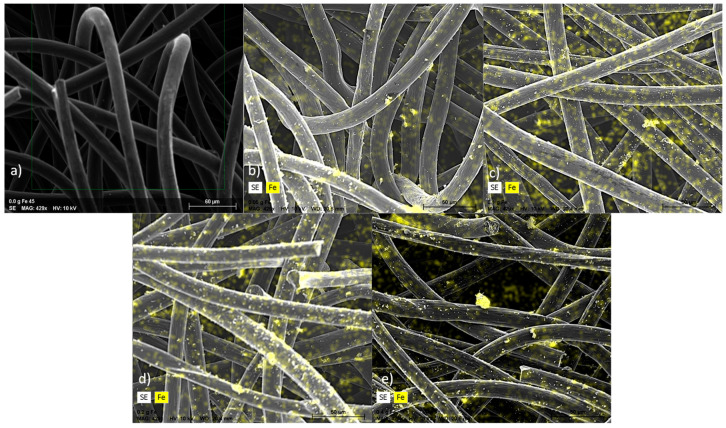
SEM/EDS analyses of anodes’ surfaces for an acceleration voltage of 15 kV (**a**) pristine, (**b**) with 0.05 g Fe_2_O_3_, (**c**) with 0.1 g Fe_2_O_3_, (**d**) with 0.2 g Fe_2_O_3_, and (**e**) with 0.4 g Fe2O3.

The photos show a pure carbon felt that was subjected to sonication (Figure 1a) and Fe_2_O_3_-modified anodes (Figure 1b–e). The mass fractions of iron that were detected (by means of a combined SEM/EDX methodology) on the electrodes were 5.5%, 6.9%, 10.7%, and 14.2% when Fe_2_O_3_ was dosed on the carbon felt in amounts of 0.05, 0.1, 0.2, and 0.4 g, respectively. Small differences in weight percentages of Fe between electrodes may be caused by limitations in the SEM/EDX technique, as this analysis shows only information on the surface of the electrode. Thus, this method was used as a qualitative confirmation of the Fe deposition, not the quantitative one. Analysis of SEM shows that iron has been deposited on the GF anode electrodes. The photos (Figure 1b–e and Figure S1) show Fe_2_O_3_ particles deposited on GF fibers. Compared to the bare electrode (Figure 1a), the fibers have a rougher surface, which significantly increases the specific surface area of the electrodes [40] and enhances and prolongs the bioadhesion of microorganisms to the surface of modified anode materials [41].

### 3.2. Electricity Generation 

**Figure 2:**
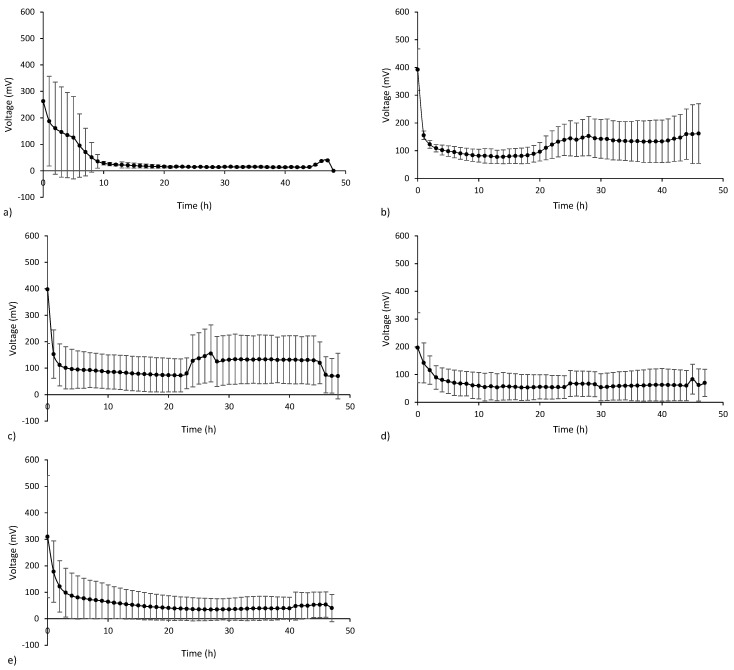
Averaged voltages (*n* = 5) from the last 5 cycles for (**a**) MFCcontrol, (**b**) MFC0.05Fe, (**c**) MFC0.1Fe, (**d**) MFC0.2Fe, and (**e**) MFC0.4Fe.

In all tested MFCs, regardless of the iron dose, the voltage was generated from the first cycle of operation. The highest voltages were obtained immediately after the addition of the substrate, which may have resulted from rapid proton flow through the membrane, but during the first 2–3 h, the voltages dropped rapidly. In MFC_0.05Fe_ (Figure 2b) and MFC_0.1Fe_ (Figure 2c), the voltage decreased until c.a. 24 h of the cycle and increased afterward. The highest average voltages in the cycle (125 ± 48.5 mV) were observed in MFC_0.05Fe_ and were about 3 times higher than in the control. The average voltages obtained in MFC_0.05Fe_ and MFC_0.1Fe_ were significantly higher than those in the other MFCs (Figure S2). In the control, MFC_0.2Fe_, and MFC_0.4Fe_, the voltage gradually decreased during the cycle to 20–50 mV at the cycle end (Figure 2). The voltage was lowest in MFC_control_, and was also the least stable, as indicated by large standard deviations. Zheng et al. [21] added Fe_3_O_4_ to MFC with a medium. Their studies showed that the output voltage at the highest dose of 18 g/L was the lowest of all MFC tested. This suggests that the voltage shifts at higher doses could be due to the accumulation of Fe occupying the attachment site of electroactive bacteria. SEM/EDX analysis showed that the anode electrodes with higher iron doses had greater roughness. High electrode surface roughness increases the likelihood of polymerization and fouling of the electrode surface as well as electrode poisoning after prolonged use [25], which can lead to lower output voltages. High Fe concentrations can have an inhibitory effect on energy generation by reducing substrate biodegradation [23,42] or by increasing internal resistance of MFC, which was confirmed in our studies (internal resistance was higher at doses of 0.2 and 0.4 than at 0.05 and 0.1 g Fe_2_O_3_/the entire surface of the electrode). Power output over 18 cycles is shown in Supplementary Materials Figure S3.

The electrochemical behavior of the control and Fe-modified electrodes with and without biofilm was tested by cyclic voltammetry in a working solution. Figure S4 shows the cyclic voltammograms recorded for all examined electrodes over a potential range of −0.50 to 0.50 V vs. SCE, which were obtained at room temperature with a sweep rate of 20 mV/s. The voltammograms of Fe-modified electrodes without biofilm did not exhibit any well-defined cathodic and anodic features related to iron behavior. However, the current of the cathodic peak corresponding to the hydrogen evolution reaction increased slightly with the addition of Fe. Nevertheless, the current increase was not strictly correlated with the amount of added iron, which could be caused by insufficient electric contact between the base electrode and the deposited iron oxide. On the other hand, after biofilm formation, the current density of the Fe-modified electrodes was significantly higher than that of the control with biofilm and the Fe-modified electrodes without biofilm. Additionally, the current values positively correlated with the amount of Fe that was deposited (188, 152, 51, and 43 mA with 0.4, 0.2, 0.1 and 0.05 g of Fe).

Furthermore, the CV profiles of the electrodes with 0.4 and 0.2 g of Fe_2_O_3_ exhibited two broad reversible features centered at −0.32 and 0.38 V vs. SCE. Therefore, those anodic peaks are most likely related to the process of iron oxidizing to Fe(II) and (III), while the cathodic peaks are associated with the reduction of those oxidized species [43]. Therefore, the changes observed after biofilm formation are most likely due to the following phenomena: a better electrical connection between iron oxide particles and the base electrode provided by the biofilm and the increased biofilm formation at higher doses of Fe. The areas under the CV curves (Figure S2b obtained in MFC_0.2Fe_ and MFC_0.4Fe_) were larger than in the other MFCs, which may indicate a higher charge capacity. This may be due to the fact that the charge capacity of the electrode is proportional to the electrode surface, which indicates that the surface area increases after modification [44], which is also visible in SEM images.

**Figure 3:**
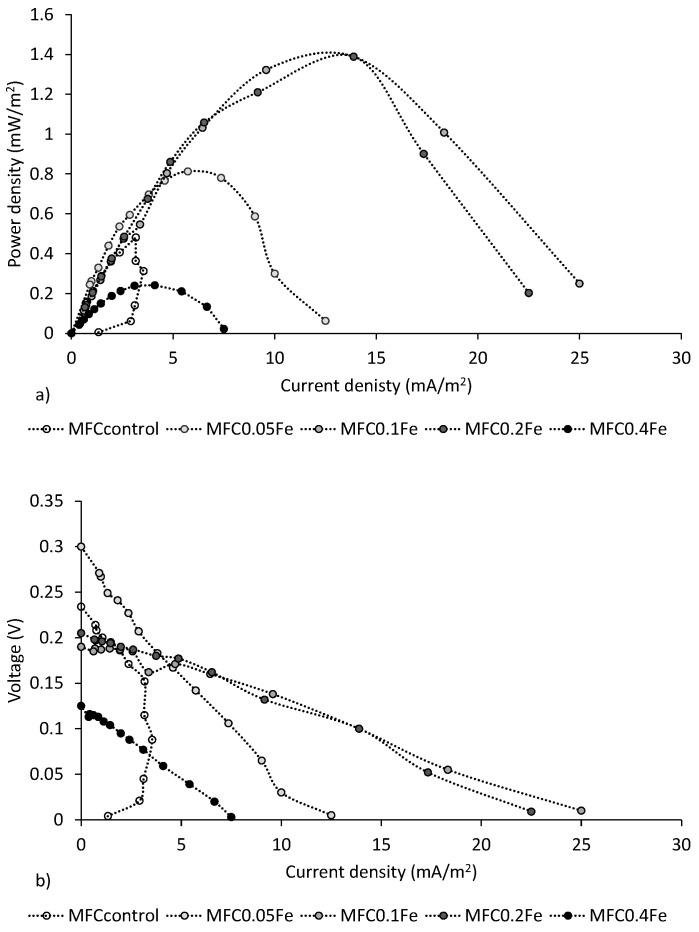
Power curves (**a**) and polarization curves (**b**) for all MFCs.

Figure 3a shows the power curves from the 14th cycle of MFC operation. The highest power density of 1.39 mW/m^2^ was obtained in MFC_0.1Fe_ and MFC_0.2Fe_. This power density was 2.8 times higher than that obtained in MFC_control_, and 1.7 and 5.8 times higher, respectively, than the values obtained in MFC_0.05Fe_ and MFC_0.4Fe_. Yang et al. [45] studied an anode with a carbonized *Shewanella* sp. biomass that produced a nanocomposite iron oxide/carbon catalyst. Their results showed that coating the anode with the nanocomposite increased the cell’s power 3.5 times compared to that of a cell with a pure CF anode. The power was higher in the cell with the modified anode because, compared to the control, the surface roughness of the anode was higher with lower charge transfer resistance. Liu et al. [23] found that, in an MFC fed with sodium acetate and increasing iron doses in the substrate, the cell power density decreased from 0.95 W/m^2^ at 200 μM Fe(III) to 0.59 W/m^2^ at 2000 μM Fe(III). In MFC_0.4Fe_ in our study, with the highest dose of Fe_2_O_3_ used for anode modification, the highest power achieved in the cell was two-fold lower than the value achieved with the control anode. The low power in MFC_0.4Fe_ could be due to an excess of iron at the anode that decreased the quality of the anode. Yang et al. [45] observed that Fe(III) aggregated on the anode can compete for electrons with the anode and inhibit the electrochemical activity of biofilms. 

The polarization curves showed that modification of the anode with iron reduced the cell’s internal resistance, most likely via better electron transfer due to the presence of iron particles. The highest internal resistance of 1029 Ω was recorded in MFC_control_, while the lowest internal resistance (184.9 Ω) was recorded in MFC_0.1Fe_ (Figure 3). Increasing the iron dose used for anode modification from 0.1 to 0.4 g gradually increased the internal resistance of the cell to 397 Ω in MFC_0.4Fe_. In a study by Mohamed et al. [6], electrode position of iron (200 mM FeCl_3_ working solution) on CF, G, and CC electrodes caused the total internal resistance of an MFC to decrease by 2.0, 1.9, and 1.4 times, respectively. In addition, electrolytic deposition improved the wettability and increased the porosity and biocompatibility of the surfaces. 

### 3.3. COD Removal

The elimination of organic compounds in the MFC is carried out by their microbial decomposition, the products of which are carbon dioxide, hydrogen protons, and electrons, the acceptor of which is the electrode. The decomposition of organic compounds can be illustrated by the example of acetic acid as follows:

C_2_H_4_O_2_ + 2H_2_O → 2CO_2_ + 8H^+^ + 8e^−^

Complex organic compounds must be converted to monosaccharides or other low-molecular-weight compounds [46]. Therefore, the high biodiversity of microorganisms is important in MFC systems using complex effluents. Organic nitrogen in wastewater is converted to NH_4_-N, which is removed in the MFC mainly by migration through the EMF and volatilization in the cathode chamber [47] or by precipitation in the form of struvite, cattiite ((Mg_3_(PO_4_)_2_·22H_2_O)) or in the presence of iron-vivianite (Fe_3_(PO_4_)_2_·8H_2_O) [48]. In anaerobic environments, in the presence of Fe(III), NH_4_^+^ is an electron donor and is oxidized to NO_2_^−^ by reduction of Fe(III) to Fe(II) according to the formula [49] (Equation (1))

3Fe_2_O_3_·0.5H_2_O +10H^+^ + NH_4_^+^ → 6Fe^2+^ + 8.5H_2_O + NO_2_^−^ (ΔGr ≤ 145.08 kJ/mol) (1)

**Figure 4:**
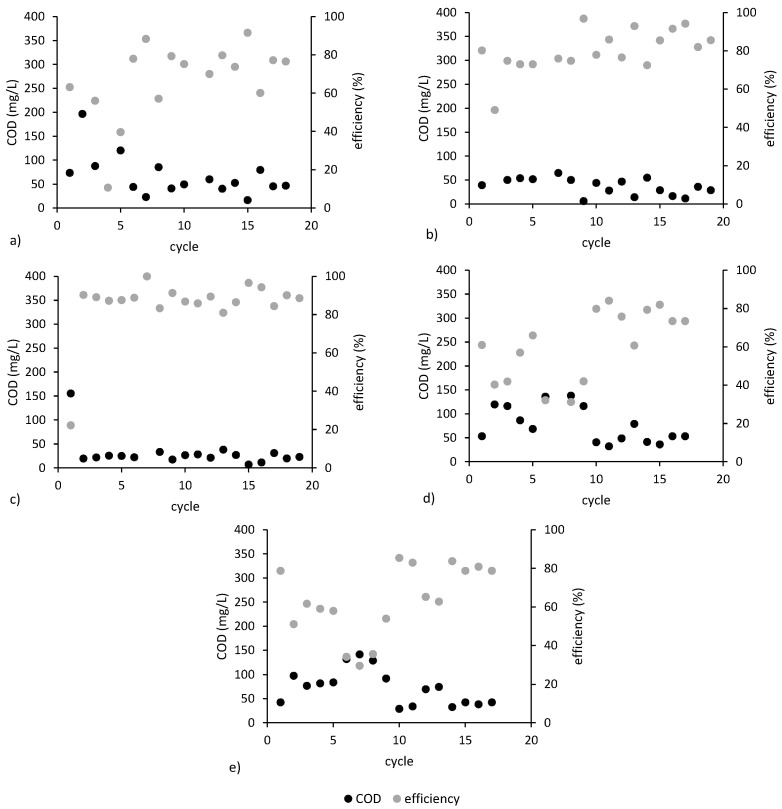
COD concentration in the effluent and COD removal efficiency in (**a**) MFC_control_, (**b**) MFC_0.05Fe_, (**c**) MFC_0.1Fe_, (**d**) MFC_0.2Fe_, and (**e**) MFC_0.4Fe_.

The average COD removal efficiency was above 70% for all reactors, indicating that the anaerobic biofilm on the anode was well developed and active. Low iron dosages favored more stable and effective COD removal compared to MFC_control_ (Figure 4a). In MFC_0.05Fe_ and MFC_0.1Fe_, COD was stably removed with efficiencies of 83.96 ± 10.1% and 85.5 ± 14%, respectively (Figure 4b,c). The mean COD concentrations in the treated wastewater from MFC_0.05Fe_ and MFC_0.1Fe_ were 29.5 ± 14.9 and 30.8 ± 7.6 mg COD/L, respectively, and were significantly lower than concentrations in the other MFCs. Better COD removal can be explained by the iron-modified anode having a higher surface area, which results in better biocompatibility and adhesion of microorganisms [4,50]. However, our results suggest that if the Fe dose is too high, the stability of COD removal decreases (Figure 4d,e). For example, in MFC_0.4Fe_, the range of COD in wastewater was between 29 and 141 mg/L. Mohamed et al. [51] reported that Fe(III) at a dose of 40 mg/L in wastewater treatment systems decreases COD’s removal efficiency. At high concentrations of Fe(III) in the environment, Fe(III) penetrates bacterial cells in large numbers and reduces enzyme activity or generates toxic free radicals that damage cell structures [52,53].

The addition of iron facilitated the maintenance of a neutral pH in the MFCs. The mean pH of the effluent from MFC_control_ (pH 8.3) was significantly higher than that of the MFCs with iron-modified anodes (pH 7.7, 7.9, 7.8, and 7.8, for MFC_0.05Fe_, MFC_0.1Fe_, MFC_0.2Fe_, and MFC_0.4Fe_, respectively). Although the pH was lower in the MFCs with iron-modified anodes, it increased during the cycle, indicating that the rate of transport of protons through the PEM was slower than the rate of their production in the anode chamber. The efficiency of an MFC is optimal when a constant pH is achieved in the anode chamber, and the rate of proton production in the anode chamber is equal to the rate of their consumption at the cathode [54]. At the same time, the effluents from MFC_0.2Fe_ and MFC_0.4Fe_ had significantly lower alkalinity than that from MFC_control_. The oxidation of organic compounds usually produces more protons than bicarbonate ions, which lowers the pH and alkalinity and may harm the anode biofilm [55]. The lower alkalinity that was observed in the MFCs modified with the highest Fe doses may decrease conductivity, increase internal resistance, and decrease the efficiency of electricity generation [56]. 

The efficiency of NH_4_-N removal in all MFCs was about 37%, and the NH_4_-N concentrations in the effluents did not differ significantly. 

### 3.4. Chromatographic Analyses of VFAs 

**Figure 5:**
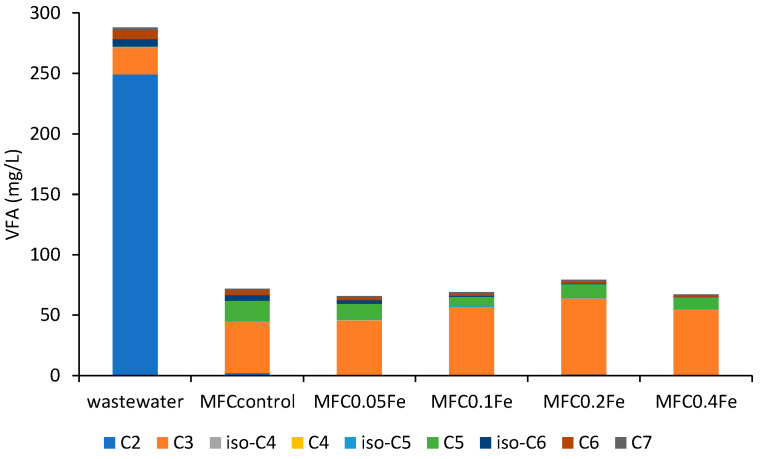
Profile of VFA concentration in raw wastewater and the effluents from all MFCs (average of *n* = 5), where C_2_—acetic acid, C_3_—propionic acid, C_4_—butyric acid, C_5_—valeric acid, C_6_—caproic acid, and C_7_—enanthic acid.

In our study, the differences in the effluent compositions indicated that the presence of Fe on the anode affected the metabolic conversions in the MFC. To illustrate the changes in VFA composition during wastewater treatment in MFCs, chromatographic analyses were performed (Figure 5). Acetic acid comprised the largest share of VFAs in the substrate (86%), while the remaining 14% were other acids, mostly propionic acid (7%). Yu et al. [57] investigated the influence of various substrates on energy production and did not note any changes in the VFA profile in an acetate-fed rector—acetic acid was the only VFA in the effluent. In our study, in contrast, acetic acid was present only in small amounts (from 0.2% in MFC_0.1Fe_ to 3.0% in MFC_control_), while propionic and valeric acid proportions significantly increased in comparison to their proportions in the raw wastewater. In the outflow from MFC_control_, propionic and valeric acids constituted 58% and 23% of VFAs, respectively, while hexanoic and iso-hexanoic acids accounted for 12%. As the iron dose was increased, the proportion of propionic acid also increased, while the proportions of valeric, caproic, and iso-caproic acids decreased. Some microorganisms are able to form storage compounds in the form of polyhydroxyalkanoate (PHA) during the anaerobic degradation of organic compounds, especially VFA. Under aerobic conditions, PHAs are used as a carbon source, e.g., during biological phosphorus removal [58]. Wang et al. [59] proposed an anaerobic pathway for the synthesis and degradation of PHAs. The type of VFA determines the composition of stored PHA, which in turn affects the types of VFA produced during PHA degradation. Polyhydroxybutyrate (one of the forms of PHA) is degraded to acetate and butyrate, and polyhydroxyvalerate (PHV) is degraded to propionate, acetate, and valerate. In our study, in the biofilm microorganisms such as *Zooglea* sp., *Dechloromonas* sp., *Acidovorax* sp., and *Hydrogenophaga* sp. (see Section 3.5), capable of PHA accumulation, were observed. Due to the prevailing anaerobic conditions, it is possible that they converted them to organic acids present in the wastewater, i.e., mainly propionic and valeric acids, which would indicate that the reserve substance was stored mainly in the form of PHV.

These results indicate that microbial metabolism in MFCs leads to the formation of VFAs other the acetic acid; this process has been observed, for example, during acetate conversion under anaerobic conditions [60]. Moreover, propionic acid that is present in wastewater may not be metabolized in MFCs. During anaerobic fermentation, the conversion of butyric acid and propionic acid to acetic acid does not occur spontaneously, due to the high Gibbs free energy of the reaction, resulting in an accumulation of the two acids [61]. The presence of propionate as a metabolic product of wastewater conversion in MFCs inhibits the activity of acidogenic bacteria and methanogens [62], which can eliminate electron loss during methane fermentation in MFCs.

### 3.5. Microbial Structure of Inoculum and Anode Biofilm in the MFCs

A total number of 498,906 readings was obtained after sequencing. The lowest number of readings was recorded in MFC_0.4Fe_ in the 10th cycle of MFC operation, and the highest in MFC_0.1Fe_ in the 10th cycle of operation (Table 1). The flattening of the rarefaction curves (Figure S6) indicates that the depth of the sequencing was sufficient. However, the OTU number for the tested samples was quite low and varied from 66 to 117. For comparison, the average number of OTUs for biofilms from the anode modified with Fe_2_O_3_ in an acetate-fed dual-chamber MFC was about 600 [63]. In our study, many unclassified microorganisms were detected, which may explain the low number of OTUs.

The ACE index in MFC_control_ and the MFCs operated with the two lower iron doses increased with time, but in the reactors with modified anodes, the diversity slightly decreased during the 10th cycle of reactor operation (Table 1). In MFC_0.2Fe_ and MFC_0.4Fe_, the ACE changed more dynamically, indicating that the presence of Fe changed the metabolism and biodiversity of the anode biofilm. In the third cycle of reactor operation (In MFC_0.2Fe_ and MFC_0.4Fe_), the ACE dropped significantly from 92 in the inoculum to about 70. However, the species diversity then increased with time, and at the end of the experiment, it was nearly two times higher than during the third cycle of MFC operation. 

**Figure 6:**
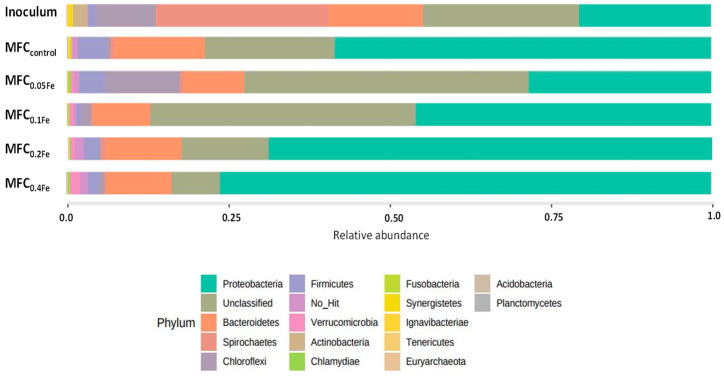
Relative abundance of particular phyla in the inoculum and anodic biomass obtained from all MFCs.

Throughout the experiment, Proteobacteria predominated in the microbial communities in the individual MFCs. The share of Proteobacteria increased in relation to the dose of iron used for the anode modification (Figure 6), reaching a value above 75% of all identified microorganisms in MFC_0.4Fe_. Proteobacteria members are electroactive bacteria crucial for extracellular electron transport [64]. They are involved in sludge hydrolysis and short-chain fatty acid production and are the main consumers of acetate, propionate, and butyrate during fermentation [65], which explains their high abundance in MFCs fed with acetate-containing wastewater. In a study in which Fe_3_O_4_ was dosed to the medium in a single-chamber MFC, the numbers of Proteobacteria in the biomass were also high, followed by Desulfobacterota and Bacteroidota [21]. The second most abundant group in the present study was Bacteroidetes (up to 11% in MFC_0.05_). A high percentage of unclassified bacteria (up to 44% in MFC_0.05_) indicated that many yet undiscovered bacteria played an important role in energy production in MFCs.

**Figure 7:**
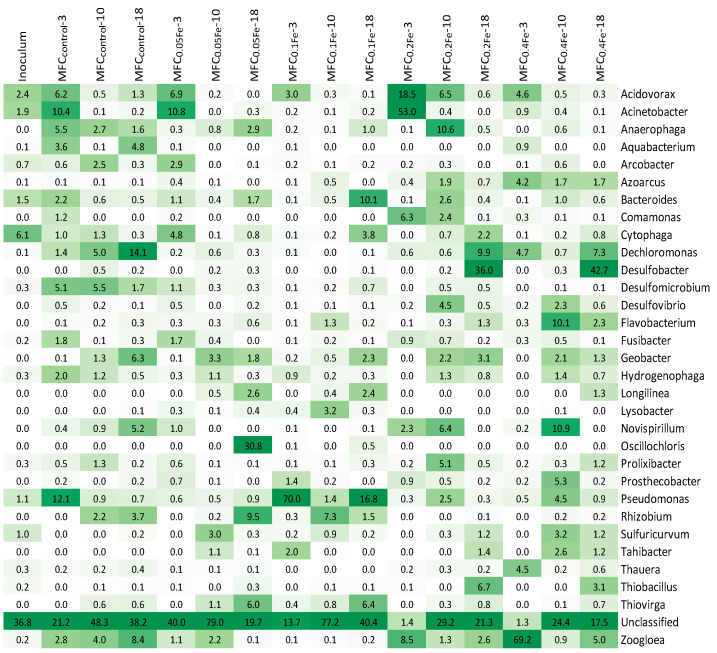
Heatmap showing 30 of the most numerous genera in the investigated MFCs, the number after the reactor name indicates the cycle in which sampling was performed.

A common feature of the microbiome in all MFCs was the presence of *Acidovorax* sp. during reactor start-up (Figure 7). Its share ranged from 3 to 18.5% depending on the MFC, and then, in subsequent cycles, it decreased to <0.5%; only in the control did its share amount to 1.3% in the 18th cycle. *Acidovorax* sp. are capable of efficient extracellular polymeric substances (EPS) production (over 150 mg/L in R2A agar medium [66]), and probably participated in anode colonization and the formation of an anode biofilm in the initial stage of MFC operation. 

One of the most frequently identified EEs is *Geobacter* sp., which is able to convert energy from the decomposition of organic compounds with electron transfer to the anode using nanowires [67]. Liu et al. [22] observed a decrease in the share of *Geobacter* sp. in the bacterial community with an increasing concentration of FeCl_3_ in a medium from 200 to 1000 µM. Zheng et al. [21] investigated the effect of Fe_3_O_4_ added to the medium in a single-chamber MFC. Their results show that the lowest tested dose of 4.5 g/L led to an enrichment of the anode biofilm in *Geobacter* sp. (17.4% for control and 31.5% for MFC with the tested Fe_3_O_4_ dose), but higher doses decreased their share. Our study shows that *Geobacter* sp. was present in all tested MFCs, with the highest proportion (6.3%) being observed in the MFC_control_. In the remaining MFCs, the share of *Geobacter* sp. ranged from 1.3 to 3.3%. The results indicate that the percentage of *Geobacter* sp. in the biofilm did not directly translate into energy production in MFCs, which was highest in MFC_0.1Fe_. The numerical abundance of microorganisms in biofilms cannot be assumed a priori to correlate to the capacities of these species to produce power [68]. In MFCs with lower *Geobacter* sp. abundances, a greater role was played by the synergistic interactions between microorganisms. In co-cultures with EE microbes, other bacteria may facilitate the current generation by removing chemicals or by producing a substrate for the current generation [69]. The lower proportion of *Geobacter* sp. in the presence of iron ions may result from the fact that these microorganisms do not appear to significantly reduce crystalline forms of Fe(III) [70]. Growing these microorganisms in media with crystalline forms of Fe(III) led to the enrichment of methane-producing cultures, while the addition of weakly crystalline Fe(III) resulted in the successful enrichment of *Geobacter* sp. [67]. The other study focused on the analysis of protein expression in *Geobacter sulfurreducens* showed that in the presence of Fe(III) oxide, about 76% of proteins were less abundant than in the presence of Fe(III) citrate due to the slower rate of bacterial metabolism and growth with an insoluble electron acceptor. Most of these proteins were involved in metabolic processes such as electron transport (13 c-type cytochromes) or were structural proteins for electrically conductive pili (PilA, [71]). 

As environmental conditions in MFCs with iron-modified anodes did not favor *Geobacter* sp., in these MFCs, electricity generation was supported by other microorganisms. At two lower doses of iron on the anodes, EEs belonging to the genera *Pseudomonas* and *Oscillochloris* were identified in large numbers. *Oscillochloris* sp. is a type of filamentous bacteria, classified as anoxygenic phototrophs, that binds carbon through the reductive pentose phosphate cycle [72]. Phototrophic bacteria of various types are capable of generating energy [73,74,75]. In the MFC_0.1Fe_, *Pseudomonas* sp. comprised up to 70% of bacteria in the anode biofilm. *Pseudomonas* sp. produces several electrochemically active phenazine derivatives, such as phenazines-1-carboxylic acid, pyocyanin, oxychlororaphin, and pyorubin, which can act as redox mediators in the MFC [76]. The high abundance of *Pseudomonas* sp. in MFC_0.1Fe_ may explain the high power obtained in this cell because electrons produced by other bacteria could be transferred to the anode via mediators produced by *Pseudomonas* sp. In high concentrations, the mediators produced by *Pseudomonas* sp. can be toxic to other species, thus giving *Pseudomonas* sp. an environmental advantage in competition with other bacterial species [69]. The energy production by pure cultures of *Pseudomonas* sp. is lower than that of biofilms with a high proportion of *Geobacter* sp.; however, this lower energy production by Pseudomonas sp. may be compensated by their high abundance in biofilm. Moreover, competition in biofilms involves many factors, and the ability to conduct current may not be the only or main reason for the predominance of high-power-producing bacteria. For example, some EEs bacteria in a mixed culture of the anode in MFC are capable of higher power production than after isolation from this biofilm and cultivation in the pure culture [68]. 

In MFC_0.05Fe_ and MFC_0.1Fe_, *Rhizobium* sp., *Thiovirga* sp., *Longilinea* sp., and *Bacteroides* sp. occurred in the anode biofilm, which all have been reported to be involved in energy generation in MFCs. The *Rhizobium anhuiense* strain is an effective EE and generated a maximum voltage of 635 mV and output power of 1.07 mW/m^2^ in a glucose-fed dual-chamber MFC operated in an open circuit [77]. *Thiovirga* sp. is a type of chemolithoautotrophic bacteria, and in MFCs, they play an essential role in sulfide metabolism and COD removal [78]. *Thiovirga* sp. shows good resistance to high metal concentrations; however, our study indicates that, although Fe presence favors *Thiovirga* sp. growth in anodic biofilm, this genus prefers lower Fe concentrations in the environment [79,80]. *Longilinea* sp. decomposes hydrocarbon into acetate and H_2_ [81]. Yang et al. [82] reported that the presence of Fe_2_O_3_ at the anode promoted the reproduction of *Longilinea* sp., which could release intracellular electrons through the metabolism of substrates or intermediates [83]. *Bacteroides* sp. were one of the main bacteria producing electricity in the constructed wetland MFC [84]. It efficiently produced electricity by degradation of organic substances, preferably acetate [85], as well as by transferring electrons that reduce Fe(III) [86].

At the two highest doses of iron at the end of the study, biomass was mostly occupied by *Desulfobacter* sp., *Dechloromonas* sp., and *Thiobacillus* sp. *Dechloromonas* sp. have been reported to both generate electricity in MFCs [87,88] and play an important role in the conversion of ferrous ions in Fe-contaminated soils or waters [89,90]. Li et al. [91] observed that during municipal wastewater treatment in MFC coupled with an up-flow in denitrification biofilter abundance of *Dechloromonas* sp. ranged from 1.29% to 9.52%. The authors reported a negative correlation between the abundances of *Dechloromonas* sp. and *Geobacter* sp., which play a predominant role in denitrification and anodic respiration. This observation indicates their similar respiring and metabolizing processes and, thus, potential competing relationships. Our studies indicate that their abundance also depends on the iron content in the environment. *Desulfobacter* sp. are a type of EE [92] using acetate as an electron donor and sulfate, sulfite, and thiosulfate as final electron acceptors [93]. *Desulfobacter* sp. generated electric power of 370–1400 mW/m^2^ in the concentration range from 550 to 1270 mg COD/(L∙h) [94]. *Thiobacillus* sp. was present in both the anode and cathode biofilm [95,96] with abundances in anode biofilms as high as 24.5% during the treatment of wastewater with high sulfur content [97]. 

In MFC_0.4Fe_ at the beginning of the experiment, Proteobacteria constituted as much as 96% of the entire bacterial community, and the *Zoogloea* sp. share was close to 70%. The predominance of *Zoogloea* sp. in the biofilm from MFC_0.4Fe_ may result from the fact that these microorganisms can produce large amounts of EPS. Production of protein- and polysaccharide-rich EPS plays a vital role in cell protection under metal stress conditions [98,99], as functional groups from EPS form complexes with metal ions protecting the cell surface [100]. Metal ions may also be sorbed on the cell surface of *Zoogloea* sp. by electrostatic interactions such as van der Waals forces [101,102]. A decrease in the abundance of *Zoogloea* sp. in the course of the experiment indicated bacterial succession maturation of the anodic biofilm. Another bacterial group that preferred high iron concentrations was *Flavobacterium* sp. (up to 10.1% of the bacterial community in MFC_0.4Fe_). Previous research indicated that *Flavobacterium* sp. predominated in acetate-fed MFC [103] and was an important player in electricity production [104,105]. 

The presence of Fe_2_O_3_ on the anode was positively correlated with the abundances of *Methylovresatilis* sp. and *Fluviicola* sp., whose share on the MFC_0.4Fe_ anode reached 1.2% and 1.5%, respectively (Figure S5). *Methyloversatilis* sp. is a type of facultative methylotroph that can grow on a variety of C_1_ and multi-carbon compounds [106] and has been reported in MFCs [107]. Our study indicated for the first time that the presence of *Methyloversatilis* sp. in the environment is stimulated by increasing Fe concentrations. It may be explained by the important role of Fe-containing enzymes in *Methyloversatilis* sp. [108] as well as by the involvement of this genus in iron conversions in the environment [109]. The positive effect of the addition of ferrous ions (FeII) on *Fluviicola* sp. abundance was observed in up-flow anaerobic sludge blanket digestion reactors in which these bacteria conducted NO_2_ reduction together with Fe(II) [110].

## 4. Conclusions

Bioelectrochemical systems that recover energy from wastewater and waste can play an important role in the energy industry’s future. In this study, to improve electrical power generation in MFCs, various doses of Fe_2_O_3_ were used to modify the anode surface. In MFC_0.1Fe,_ the power and cell resistances were the best, 2.8 times higher and 5.6 times lower than in the control. Organics removal from wastewater was more stable in MFCs with anodes modified with lower Fe_2_O_3_ doses. Although the main source of carbon in wastewater was acetate, propionic and valeric acids predominated among the VFAs in the MFC effluents; the proportion of propionic acid to other VFAs increased with increasing iron dose. EPS producers, such as *Zoogloea* sp. and *Acidovorax* sp., were abundant during reactor start-up, facilitating microbial colonization of the anode and the development of anode biofilms. The addition of iron increased the diversity of the bacterial community. Our study indicated that EE composition was strongly affected by the presence of iron on the anode. The abundance of EE *Geobacter* sp. was 2–3% on the anodes modified with Fe_2_O_3_ in comparison with 6% in the control MFC. At the two lower doses of Fe_2_O_3_, the abundance of EEs belonging to *Oscillochloris* sp. and *Pseudomonas* sp. increased, while at the two highest Fe_2_O_3_ doses, EEs belonging to *Dechloromonass* sp. and *Desulfobacter* sp. predominated in the biofilm. To improve the operation of the MFC, more research is needed to evaluate the effects of iron dose on the anode electrode, because too much iron can negatively affect the current generation and the anode biofilm, and researchers often focusing on the modification itself, ignoring the microbiological aspect. This research could help scientists further develop MFCs through low-cost methods, but more resistant materials need to be developed for industrial applications, as the technologies need to work in the long term.

## Figures and Tables

**Table 1:** Alpha diversity indicators in all MFC reactors; the number after the reactor name indicates the cycle in which sampling was performed; OUT—operational taxonomic unit, Chao1—richness estimator, Shannon—biodiversity index, ACE—abundance-based coverage estimator.

Reactor—Cycle Number	OTU	Chao1	Shannon	ACE	Total Read Counts
Inoculum	91	91.5	2.51	92.7	26,877
MFC_control_—3	103	103.6	3.14	103.6	25,053
MFC_control_—10	108	108.5	2.97	108.2	29,183
MFC_control_—18	112	114.3	2.85	114.8	28,548
MFC_0.05Fe_—3	108	108.5	2.85	108.3	35,811
MFC_0.05Fe_—10	105	108.0	1.51	108.1	38,831
MFC_0.05Fe_—18	111	111.0	2.77	111.3	24,160
MFC_0.1Fe_—3	109	110.6	1.47	111.1	43,390
MFC_0.1Fe_—10	101	101.6	1.23	102.8	49,705
MFC_0.1Fe_—18	117	117.9	2.69	118.5	33,249
MFC_0.2Fe_—3	66	69.0	1.72	68.5	24,281
MFC_0.2Fe_—10	101	101.5	3.14	103.5	28,223
MFC_0.2Fe_—18	118	122.7	2.57	122.5	30,545
MFC_0.4Fe_—3	64	65.3	1.45	66.8	28,970
MFC_0.4Fe_—10	103	104.1	3.21	105.3	17,967
MFC_0.4Fe_—18	111	111.8	2.56	113.0	34,113

## Data Availability

The datasets used and/or analyzed during the current study are available from the corresponding author upon reasonable request.

## Supplementary Materials

The following are available online at https://www.mdpi.com/article/10.3390/ijerph20032580/s1, Figure S1. SEM/EDS analyses of anodes’ surfaces for an acceleration voltage of 15 kV: (a) pristine, (b) with 0.05 g Fe_2_O_3_, (c) with 0.1 g Fe_2_O_3_, (d) with 0.2 g Fe_2_O_3_, (e) with 0.4 g Fe_2_O_3_. Figure S2. Statistical differences in the voltages obtained in the individual reactors (ANOVA; Tukey’s HSD post hoc test), *p* < 0.05, * significantly higher than in the remaining MFCs. Figure S3. Power output within 18 cycles for (a) MFC_control_, (b) MFC_0.05Fe_, (c) MFC_0.1Fe_, (d) MFC_0.2Fe_, (e) MFC_0.4Fe_. Figure S4. CV for (a) abiotic anodes, (b) with biofilm. Figure S5. Refraction curves; the number after the reactor name indicates the cycle in which sampling was performed. Figure S6. Top 25 genera with abundances most strongly correlated with the dose of Fe_2_O_3_ used for anode modification.
