# Supplementary material for: Anode Modification with Fe2O3 Affects the Anode Microbiome and Improves Energy Generation in Microbial Fuel Cells Powered by Wastewater
Source: Int J Environ Res Public Health. 2023 Jan 31;20(3):2580. doi: 10.3390/ijerph20032580 (PMC9916399; doi:10.3390/ijerph20032580)
Supplement: Supplementary file 1 [file ijerph-20-02580-s001.zip › ijerph-2099857-supplementary.pdf]

Supplementary materials

Anode Modification with Fe_2O_3 Affects the Anode Microbiome and Improves Energy Generation in Microbial Fuel Cells Powered by Wastewater

Dawid Nosek [1,*], Tomasz Mikołajczyk [2] and Agnieszka Cydzik-Kwiatkowska [1]

1 Department of Environmental Biotechnology, University of Warmia and Mazury in Olsztyn, Słoneczna 45 G, 10-709 Olsztyn, Poland
2 Department of Chemistry, University of Warmia and Mazury in Olsztyn, plac Łódzki 4, 10-721 Olsztyn, Poland
* Correspondence: dawid.nosek@uwm.edu.pl; Tel. +48-89-5234144

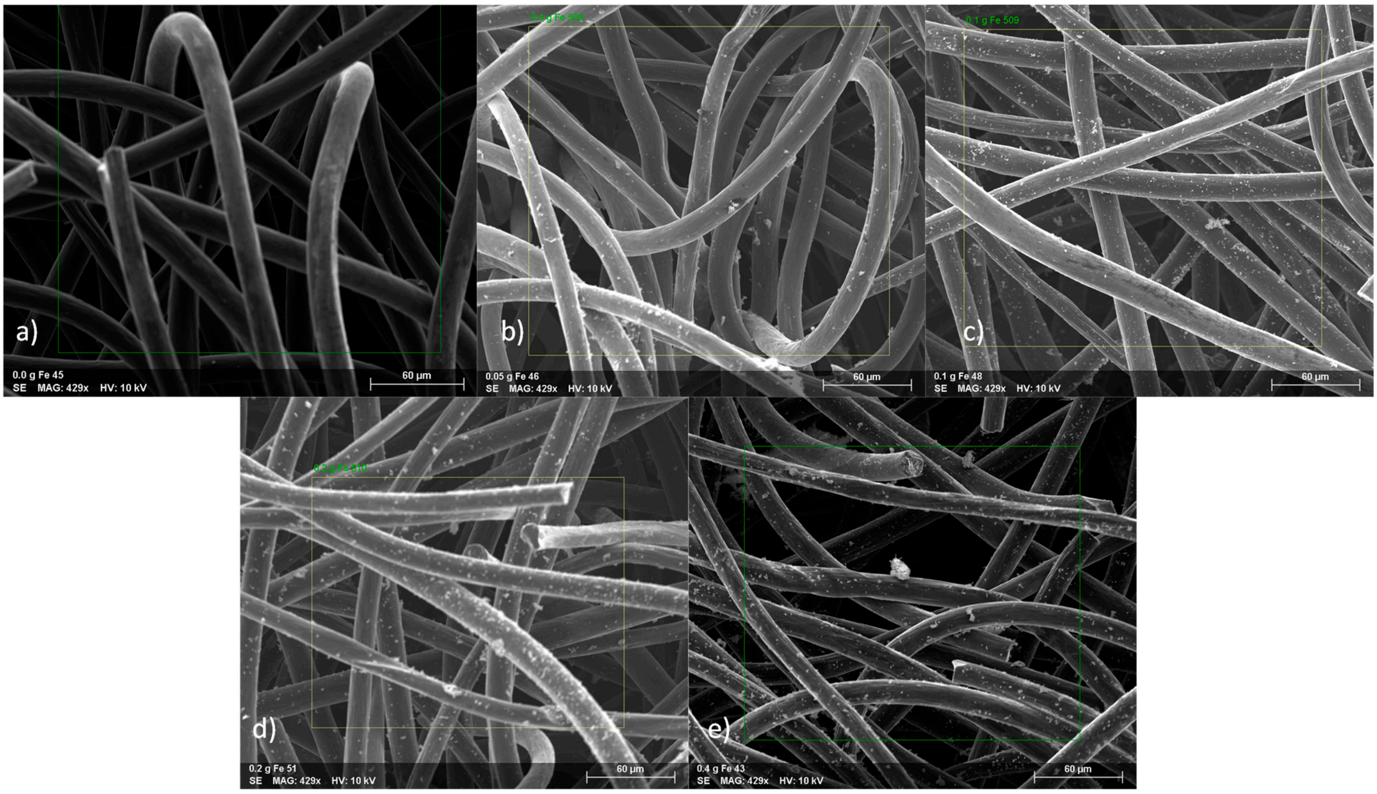

Figure S1. SEM/EDS analyses of anodes' surfaces for an acceleration voltage of 15 kV: (a) pristine, (b) with 0.05 g Fe2O3, (c) with 0.1 g Fe2O3, (d) with 0.2 g Fe2O3, (e) with 0.4 g Fe2O3.

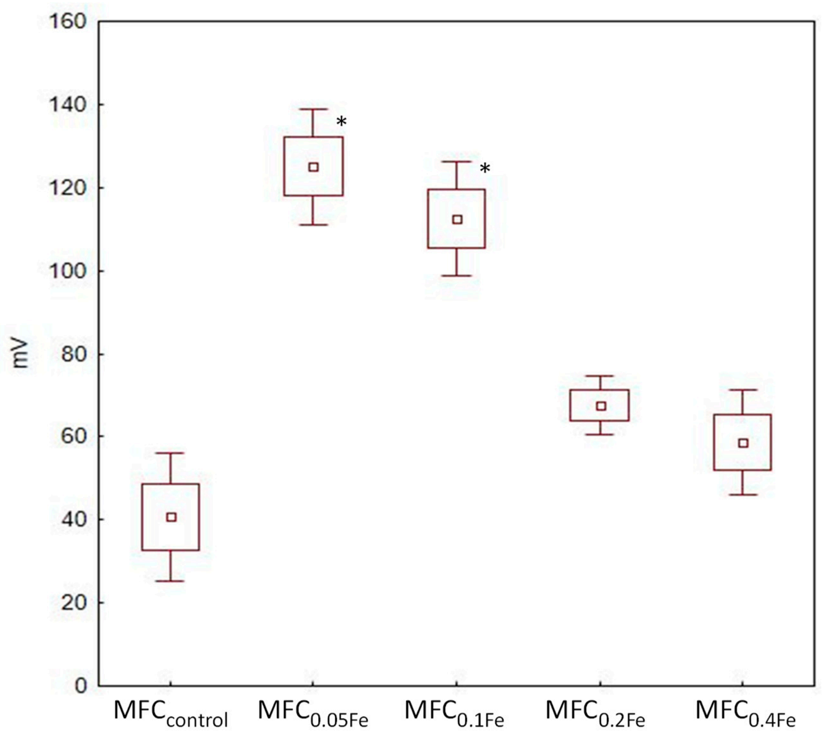

Figure S2. Statistical differences in the voltages obtained in the individual reactors (ANOVA; Tukey’s HSD post hoc test), $p<0.05$, * significantly higher than in the remaining MFCs

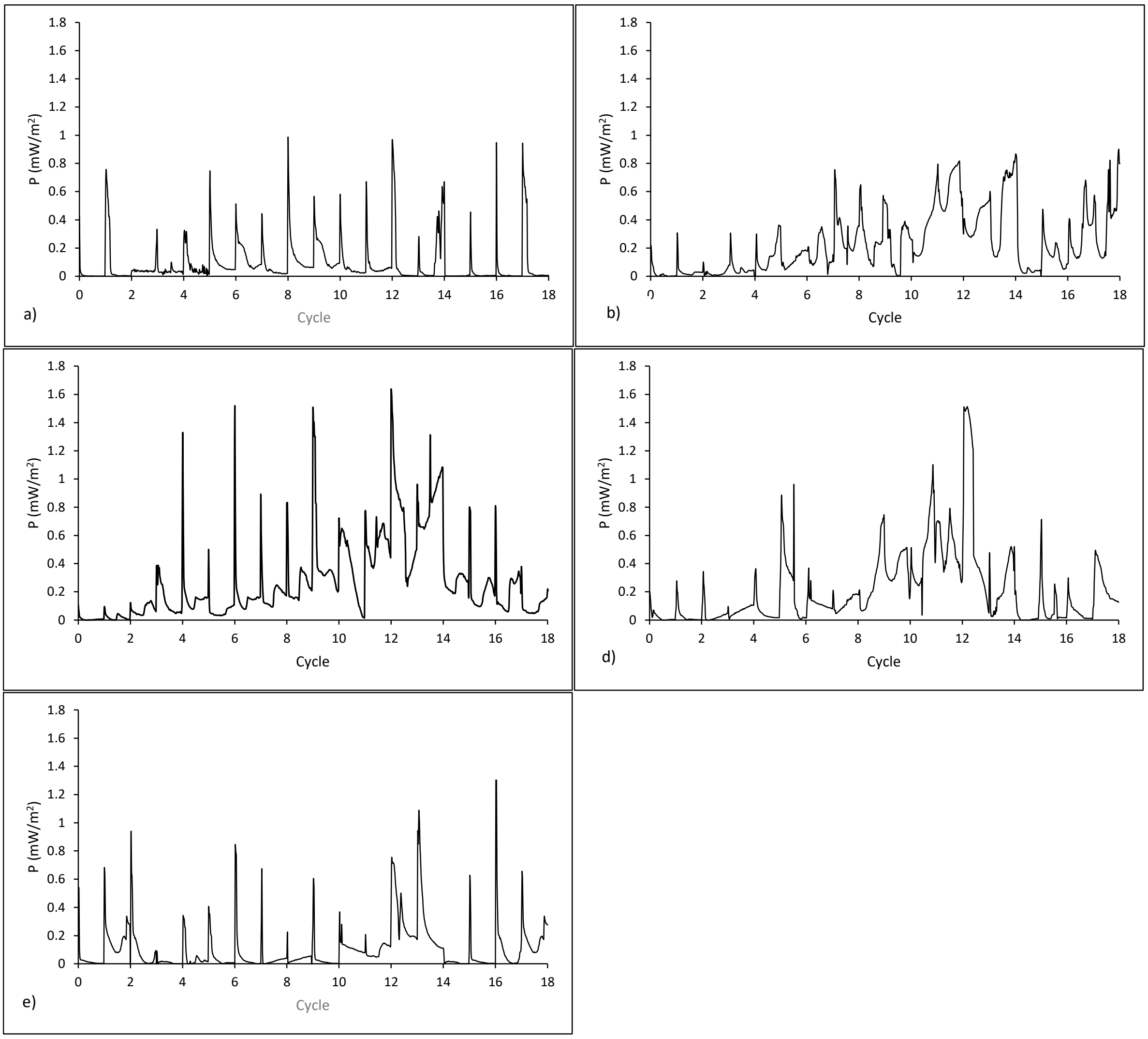

Figure S3. Power output within 18 cycles for (a) MFC$_{control}$, (b) MFC$_{0.05Fe}$, (c) MFC$_{0.1Fe}$, (d) MFC$_{0.2Fe}$, (e) MFC$_{0.4Fe}$.

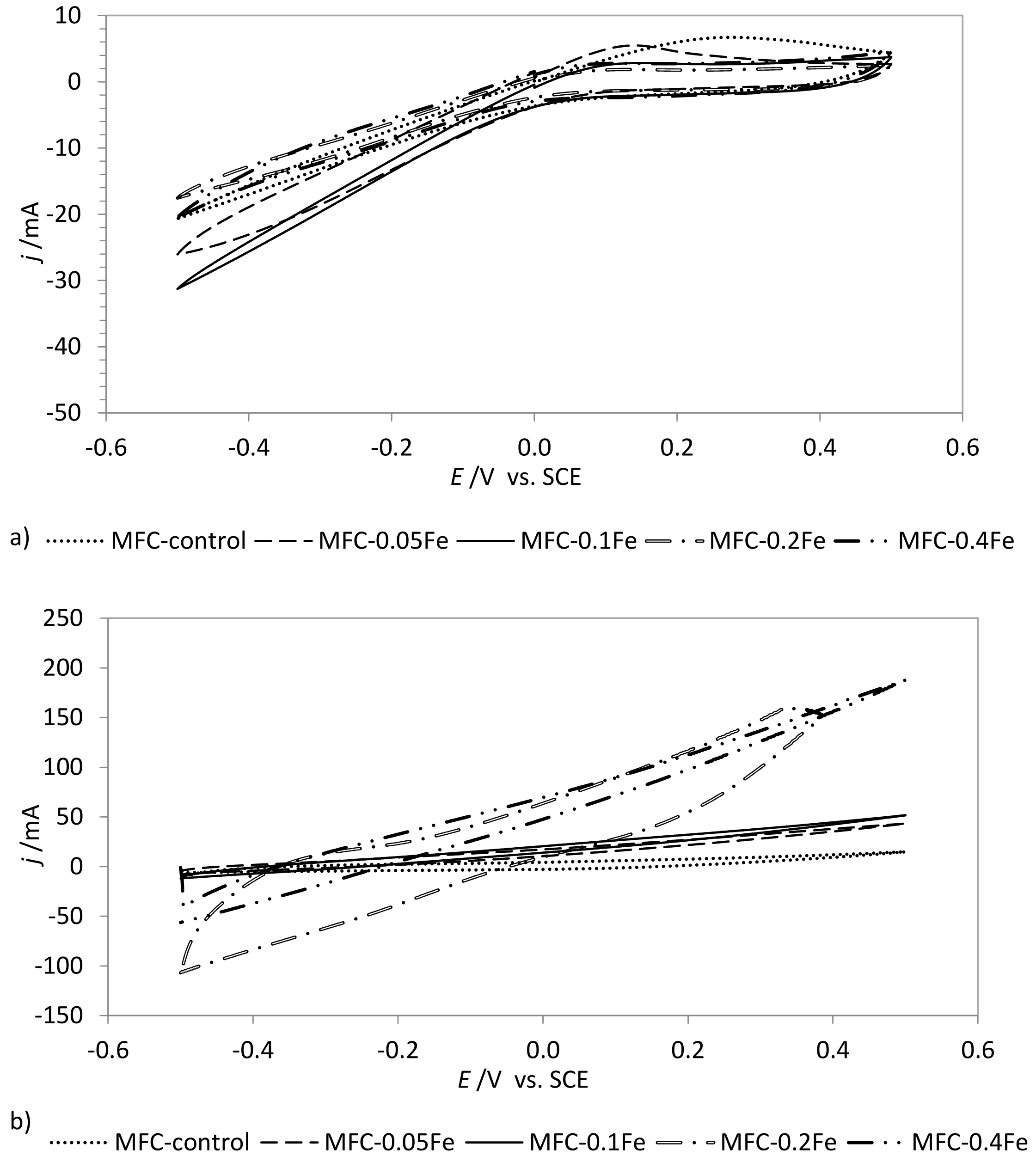

Figure S4. CV for (a) abiotic anodes, (b) with biofilm

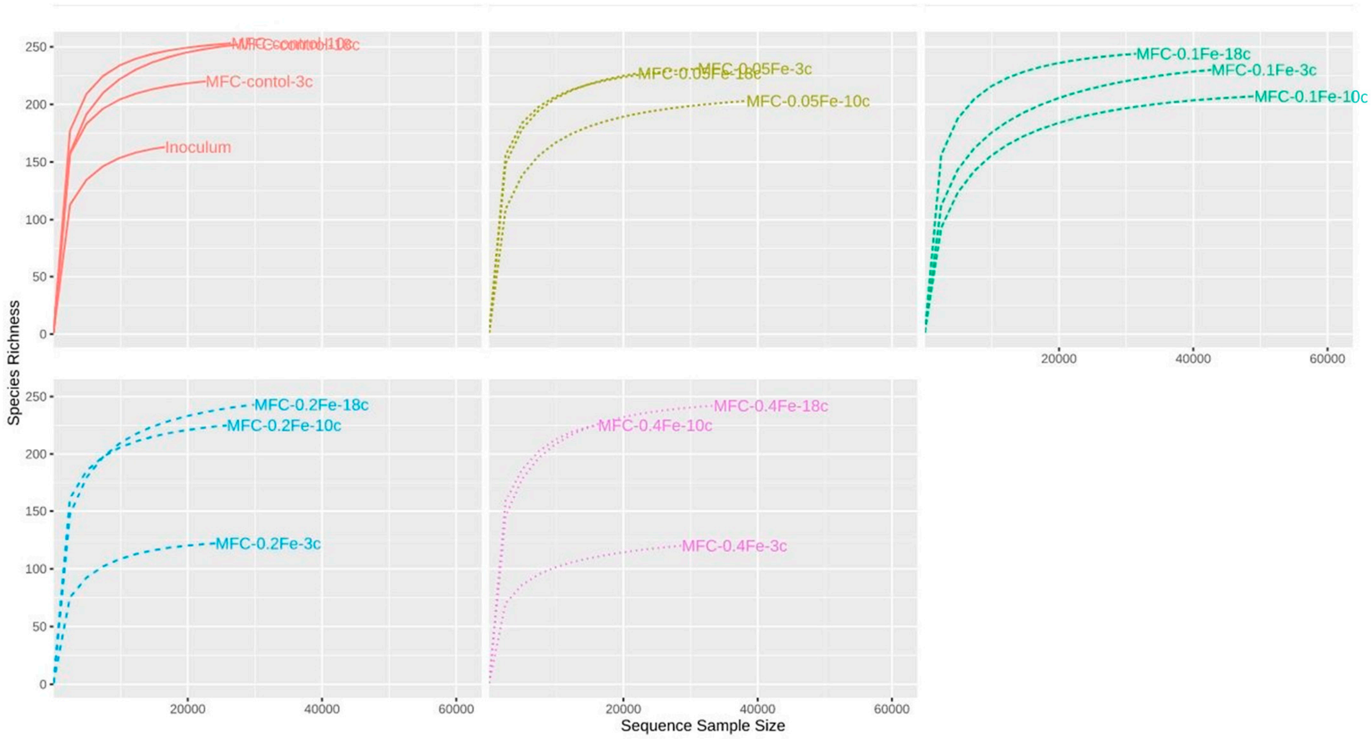

Figure S5. Refraction curves; the number after the reactor name indicates the cycle in which sampling was performed.

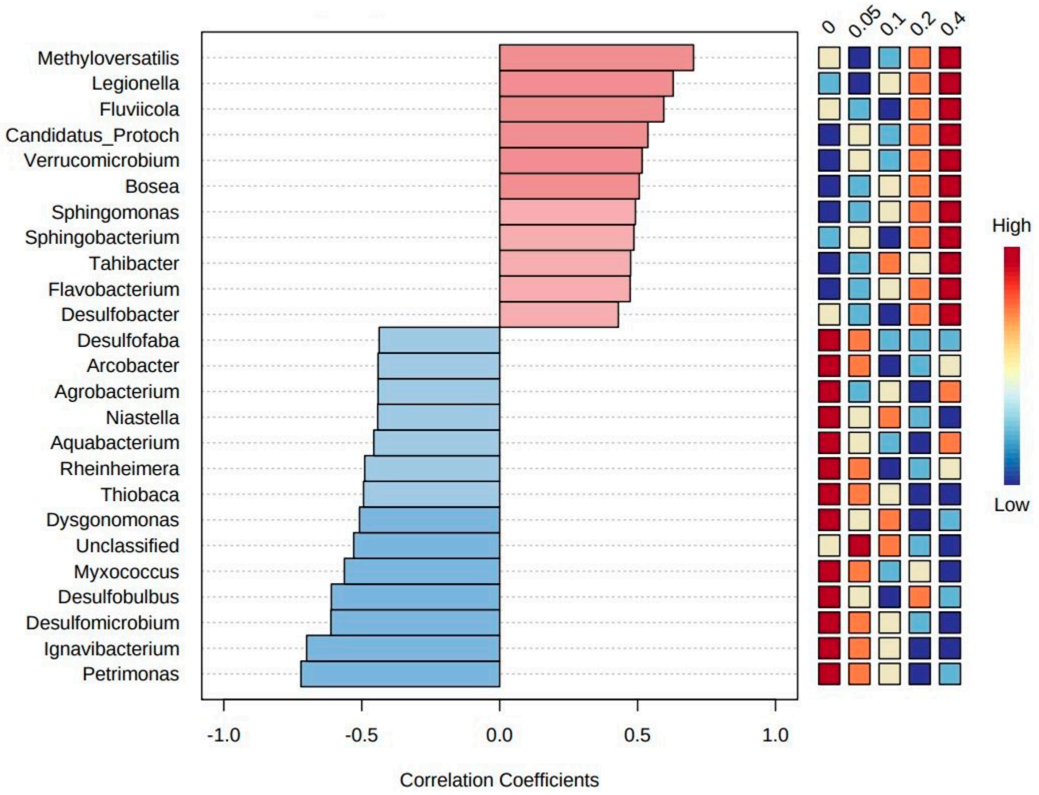

Figure S6. Top 25 genera with abundances most strongly correlated with the dose of Fe_2O_3 used for anode modification.
